# Frailty syndrome and risks for falling in the elderly community

**DOI:** 10.1590/2317-1782/20212021025en

**Published:** 2022-08-08

**Authors:** Carlos Kazuo Taguchi, Pedro de Lemos Menezes, Amanda Caroline Souza Melo, Leonardo Santos de Santana, Wesley Rayan Santos Conceição, Gabrielle Feitosa de Souza, Brenda Carla Lima Araújo, Allan Robert da Silva

**Affiliations:** 1 Universidade Federal de Sergipe – UFS - São Cristóvão (SE), Brasil.; 2 Universidade Estadual de Ciências da Saúde de Alagoas – UNCISAL - Maceió (AL), Brasil.

**Keywords:** Frailty, Frail Elderly, Accidental Falls, Healthy Aging Aged

## Abstract

**Purpose:**

To identify the prevalence of Frailty Syndrome in the elderly and the relationship with risk of falling.

**Methods:**

Descriptive, cross-sectional, and analytical clinical study. One hundred and one volunteers over 60 years old were submitted to audiological evaluation, Dynamic Gait Index - Brazilian brief (DGI), Timed Up and Go (TUG) and Edmonton Fragility Scale (EFE) that verified, respectively, hearing thresholds, frailty syndrome, functional and dynamic balance, and risk of falling. The simple percentual distribution, the Wilcoxon´s test and the Bivariate Correlation with Pearson's coefficient were used for statistical analysis. Limits equal to or less than 1.0 and 5.0% were adopted.

**Results:**

EFE identified 22.8% of volunteers as fragile and 22.8% as vulnerable. DGI and TUG found 34.6% and 84.1% of at risk for falls, respectively. Significant correlations between EFE and DGI (p <0.01), EFE and TUG (p <0.01), and DGI and TUG (p <0.01) were observed. Pearson's coefficient between EFE and DGI, EFE and TUG, and DGI and TUG were -0.26, -0.41, and 0.46, respectively. An association between DGI and TUG and age (p <0.01) was identified. No correlation between EFE and sex or age was found.

**Conclusion:**

Frailty and pre-frailty were identified in a significant segment of the volunteers, especially in the oldest subjects. Functional and dynamic balance were moderately correlated with frailty, which demonstrated that frailty syndrome increases the risk of falls.

## INTRODUCTION

The increased life expectancy of the global population is an urgent and irreversible fact. In Brazil, the number of elderly individuals will surpass 19.2 million (9.2%), reaching 58.2 million (25.5%) by 2060, which will in turn imply a higher demand on the health services targeted at this population^(1)^. Physiological changes occur during the aging process, to a greater or lesser extent, in all organs and systems^(2)^, which tends to result in loss of autonomy and independence. Still, there may be a greater susceptibility to diseases, functional decline and propensity to falls. This set of signs/symptoms is clinically defined as frailty syndrome^(3)^.

Fall risk assessment can be performed by several qualitative evaluation tests such as the Dynamic Gait Index -Brazilian brief (DGI)^(4)^, which evaluate dynamic balance and gait in different contexts, and the Timed Up and Go (TUG)^(5)^ test, which analyzes functional balance. Since there is no gold standard, frailty can be assessed by several tools, testing different domains and functional abilities and establishing a cut-off score adapted to the characteristics of each population group, as demonstrated in a systematic review study^(6)^.

Both the risk of falls and the frailty of elderly people are phenomena that can be prevented, monitored and referred, which makes this topic a priority for the development of protocols and services for the ageing population^(7)^. It is evident that Speech-Language Pathology and Audiology is a field crucial to the production of knowledge for geriatrics/gerontology. This highlights the need to further develop studies on aging, to be able to define interventions and lines of research for the area^(8)^. Recent evidence points to a tendency towards rehabilitation, including but not limited to prevention strategies, and also identified Audiology as the area that least contributed to such knowledge^(9)^.

Researching the association of frailty, gait and dynamic balance can provide the initial criteria for primary care-based interventions, in which Speech-Language Pathology and Audiology operates within a broad scope, encompassing prevention, education and intervention. Scientific advances do contribute to the strengthening and consolidation of this knowledge, and can reinforce the evidence that longevity and functionality are strongly related to the degree of frailty, as well as the aggravation of the condition when preventive measures and intervention strategies are not adopted^(10)^.

Based on the premise that frailty involves the complex interaction of biological, psychological and social factors throughout life^(2)^, and that the effects of this process may become irreversible, the purpose of this study was to identify the prevalence of Frailty Syndrome in the elderly and its relationship with the risk of falls.

## METHODS

Descriptive, cross-sectional and analytical clinical study, developed in the Hearing and Balance Laboratory (LAE - Laboratório de Audiologia e Equilíbrio), with volunteers recruited from the outpatient clinics of the Otorhinolaryngology, Gastroenterology and Cardiology departments of the home institution's University Hospital, and students from the partner institution's University of the Third Age. The project was approved by the Research Ethics Committee under protocol No. 10266919.1.0000.

The sample, out of expediency, was recruited between September 2019 and March 2020 from the waiting room of the abovementioned specialist offices, as well as from third age classrooms, through explanation and invitation. A total of 101 volunteers were evaluated, of which 80 were female and 21 were male, all aged 60 years or older. All participants signed the informed consent form.

Patients with impaired speech comprehension due to moderate to severe sensorineural hearing loss; with neurological or psychiatric disorders that limited the execution of the assessment tasks; or with prostheses or orthoses for structural alterations of the lower limbs were excluded from this study.

As inclusion criteria, in addition to the age variable, a basic audiological assessment was performed with investigation of the air and bone conduction threshold, as well as a logoaudiometry evaluation. The well established procedures and parameters of the clinical practice guidelines were adopted^(11)^.

### All participants underwent the following procedures:

1. Collection of identification and sociodemographic data regarding: age; education; monthly income; leisure activities; medication; dizziness complaint; self-reported falls in the last year; smoking; alcohol consumption; symptoms of other comorbidities, and self-assessment of the degree of satisfaction with their health;

2. The Edmonton Frailty Scale (EFE) is validated for Brazilian Portuguese^(12)^ and consists of 11 items in which the evaluator can mark up to three options for different answers, where a = zero (0), b = one (1) and c = two (2) points. The survey is complied with the following steps: 1) Assessment of Cognition, in which the elderly patients were asked to draw a clock marking 11:10 am; 2) General health in the last 12 months, in which the elderly patients answered the question: how many times were you hospitalized?; 3) General health status, in which the elderly patients were asked to describe their health in general; 4) Functional independence, where elderly patients listed which activities they needed help to execute; 5) Social support, in which elderly patients were asked if they could rely on the help of someone to support their needs when assistance was required; 6) Use of medicines, determining if patients routinely take five or more different types of medications and prescription drugs (prescribed by their doctor); 7) Use of medicines, where they were asked if they tend to forget to take their medications; 8) Nutrition, where they were asked if they had lost weight recently, noticing whether their clothes were increasingly loose-fitting; 9) Humor, where they were asked if they frequently feel sad or depressed; 10) Continence, where they were asked if there was a problem with lack of control of urine; 11) Functional performance, in which the Timed Up and Go – TUG^(5)^ test was performed.

In TUG testing, the patient's physical performance for dynamic balance is evaluated within a timed and defined route. This exercise consists of standing up from a sitting position, walking three meters, circumventing a static object, returning and adopting the initial position. When the time spent to complete the task is equal to or less than 10 seconds, the volunteer is classified as a low risk, between 11 and 20 seconds as a medium risk, and more than 20 seconds as at high risk of falls.

The maximum score of 17 points was adopted for the EFE test, with items 6 to 10 being 1 point each and the others up to 2 points. A score between 0 to 4 points in the EFE corresponds to the non-frail classification; from 5 to 6 the subject is considered vulnerable or in pre-frailty; from 7 to 8 indicates mild frailty; from 9 to 10 is moderate, and above 11 is severe^(12)^.

1. In order to evaluate the risk of falling, volunteers were submitted to the Dynamic Gait Index (DGI) – Brazilian brief^(4)^, composed of five tasks capable of assessing gait and functional balance. As described in Chart 1, in this test the volunteer must walk in a straight line (about three meters) to evaluate the gait in different movement contexts, such as with changes in speed, horizontal and vertical head movements, rotational motion spinning on the body's own axis, and climbing and descending stairs. The variability of the score ranges from 0 to 3, where 0 indicates severe impairment, 1 moderate impairment, 2 mild impairment, and 3 indicates normality for the task execution. The highest score is 15 points, and a test result equal to or less than 11 points indicates a high risk of falls.

**Chart 1:** Description of the five tasks of the Dynamic Gait Index – Brazilian brief (DGI)

Task 1	Gait with horizontal movements (rotation) of the head
Task 2	Gait with vertical movements (rotation) of the head
Task 3	Gait and rotation on own body axis (pivoting)
Task 4	Circumventing the obstacle
Task 5	Climbing up and down stairs

At the end of the collection the data was analyzed and possible correlations were established. For the statistical analysis the dependent variable of each individual response for each scale was considered. Descriptive measures with mean, median and standard deviation were adopted; the Wilcoxon tests and bivariate correlation with degree of linear relationship were analyzed using Pearson's coefficient, with significance level of 0.01 and 0.05 highlighted with * and ** respectively.

## RESULTS

The following results correspond to the data analysis regarding 101 elderly patients, aged between 60 and 85, with an average of 69.7 (±6.5) years. Data from 80 (79.2%) female volunteers and 21 (20.8%) male volunteers were analyzed (Table 1). From the 46 elderly patients (45.5%) identified as frail and vulnerable, 38 (82.6%) were female, and 8 (17.4%) were male. Among those who presented some degree of frailty, 12 (26.1%) presented mild frailty; 7 (15.2%) moderate, and 4 (8.7%) severe frailty.

**Table 1:** Distribution of the mean, median and standard deviation of age among the 101 elderly volunteers evaluated, according to gender

Statistics	Male	Female	General
Sample (n)	21	80	101
Mean	67.6	69.0	69.7
Median	67	69	68
Standard Deviation	6.0	6.6	6.5

After the DGI application, 34 (33.7%) of the volunteers were within the cut-off point for risk of falls. The observed variation was between 4 and 15, with an average of 12.2 (±2.3) points, as shown in Table 2. When considering the group with frailty separately, this percentage was equivalent to 16 (36.9%) elderly participants who presented risk of falling.

**Table 2:** Distribution of the mean, median and standard deviation of the results among the 101 elderly volunteers, according to the *Dynamic Gait Index*-*Brazilian brief*, *Timed Up and Go* and Edmonton Frailty Scale tests

Statistics	DGI	TUG	EFE
Mean	12.2	12.7	4.6
Median	13	12	4
Standard Deviation	2.3	3.38	2.6

**Caption:** DGI = *Dynamic Gait Index*-*Brazilian brief*; TUG = *Timed Up and Go*; EFE = *Edmonton Frailty Scale*

In Table 2, a variation from 7 to 26, with an average of 12.7 (±2.6) seconds was observed from applying the TUG. 85 (84.1%) volunteers in the sample group were at risk of falling, with prevalence among frail participants reaching 100%.

With the EFE test, the results fluctuated between 1 and 11 with an average of 4.6 (±2.6) points, which indicated variation from the vulnerable to the severely frail categories. 23 (22.8%) volunteers were identified as frail, and the same percentage as vulnerable.

The Wilcoxon test observed no correlation between EFE and gender (p=0.68), which proved that this variable alone is not a predictor of frailty.

The incidence of self-reported falls in the last year was around 22.7%, and for those with frailty syndrome this value reached 28.0%.

Table 3 presents the results of the Bivariate Correlation Test, within Pearson's linear correlation coefficient value. There was a significant correlation between the Edmonton and DGI scales (p<0.01), as well as between the Edmonton and TUG scales (p<0.01). However, the correlation was inverse and weak in the first case (r=-0.26), while in the second case the relationship was direct and moderate (0.47). The statistical study also revealed an inverse and significant correlation between the DGI and TUG scores (p<0.01), with moderate association (r=-0.41).

**Table 3:** Results of the bivariate correlation test with Pearson coefficient, according to the Edmonton Frailty Scale *Dynamic Gait Index*- *Brazilian brief* and *Timed Up and Go*

Pearson Correlation	EFE	DGI	TUG
EFE	1	-0.26^*^	0.47*
DGI	-0.26*	1	-0.41*
TUG	0.47*	-0.41*	1

* significant correlation at level 0.01

**Caption:** EFE = *Edmonton Frailty Scale*; DGI = *Dynamic Gait Index*-*Brazilian brief*; TUG = *Timed Up and Go*

Table 4 shows that, by applying the Bivariate Pearson Correlation Coefficient, age was found to be inversely, significantly and moderately associated with DGI (p<0.01 and r=-0.33), and directly, significantly and weakly correlated with TUG (p=0.01 and r=0.18). No association was found between age and EFE. An influence of TUG on the results, indicating changes in EFE with varying levels of frailty was observed. There was a significant association (p<0.01) and direct, moderate correlation (r=0.47) between both tools.

**Table 4:** Results of the bivariate correlation test with Pearson coefficient, according to Age, Edmonton Frailty Scale, Dynamic Gait Index-*Brazilian brief* and *Timed Up and Go*

Pearson Correlation	Age	DGI	TUG	EFE
Age	1	-0.33^*^	0.13**	-0.20
DGI	-0.33*	1	-0.41*	-0.26*
TUG	0.13**	-0.41*	1	-0.47*
EFE	0.20	-0.26*	-0.47*	1

* significant correlation at level 0.01;

** significant correlation at level 0.05

**Caption:** DGI = *Dynamic Gait Index*-*Brazilian brief*; TUG = *Timed Up and Go*; EFE = *Edmonton Frailty Scale*

## DISCUSSION

The DGI-Brazilian brief was able to identify that one third of the sample group scored below the cut-off point, and that with TUG about 20.0% of the sample presented low risk of falls. These results are in line with recent studies^(5,13)^, which found DGI results of 30.0 to 40.0% of elderly individuals with altered functional balance. Regarding the performance in *Timed Up and Go*, the results were in agreement with other studies^(13,14)^ that found 60.0 to 80.0% of elderly people living in the community as presenting alterations.

It is noteworthy that applying both tools was useful to estimate future falls, considering that moderate and negative correlations were obtained, as shown in Table 1, being corroborated by another study^(4)^.

The strong probability of longer-living elderly people with a higher risk of falling^(4,12)^ was in accordance with the findings obtained by the present study, in which age was moderately and weakly correlated with the DGI-Brazilian brief and TUG. Shorter time spent in TUG suggested that elderly patients may have good functional mobility, but in spite of that, a significant number of individuals with higher scores were more prone to falls, with the possibility of concomitant complaints of dizziness and imbalances also being raised^(15)^, however this was not analyzed in this research.

The prevalence of frailty observed in this study was similar to other studies that found an average EFE score between 25.9%^(16)^ and 30.9%^(17)^. On the other hand, the observed prevalence was lower than another study that found percentages between 40.5%^(18)^, with more divergent results being obtained with the same tool, showing percentages between 56.9%^(19)^ and 86%^(20)^.

This wide variation of results was recognized by a review study with meta-analysis^(6)^, which indicated a variation of 6.7 to 44.0%, which corroborated the present results. Other studies showed lower percentages than those observed here, ranging between 7%^(21)^ and 10.5%^(22)^, which would be explained by the characteristics of populations with better environmental or socioeconomic conditions, where longevity is related to better quality of life. Health-related quality of life is strongly associated with frailty^,^ and can be improved or impaired according to the socioeconomic environment or social situation of the elderly patient. A significant portion of elderly people, therefore, fall within the pre-frailty condition^(21,22)^, and are consequently included in the group that should be identified early, since they have an increased risk of frailty, ranging from 13 to 31%^(11)^. It is important to point out that this is a worrying situation because it only identifies the illness at onset, which implies a need for more preventative interventions to delay the frailty process and the possible loss of functional capacity^(23)^.

The prevalence of self-reported falls in this study was lower than that of other studies that found higher percentages of up to 51.7%^(24)^.

Despite the absence of correlation between previous falls and Frailty Syndrome, studies have shown an association between these variables^(20,24)^, which was corroborated by results similar to those obtained in the present study^(6)^.

A 28% incidence of risk of falling associated with a 74.2% incidence of Frailty Syndrome in elderly people shows the correlation between falling and frailty. It also reveals the possibility of bidirectionality, wherein falling makes the elderly person more fragile, while frailty in its turn promotes falling events^(18)^.

As previously mentioned, Speech-Language Pathology and Audiology does not view Frailty Syndrome as a potential object of study, which makes the development of this research an urgent task, given the irreversible and urgent fact of population ageing. Measures must be taken at the three levels of health promotion for the elderly, stemming from scientific evidence that shows frailty to be a predictor for the risk of falls^(24)^.

The absence of a correlation between EFE and gender differ with research reviews that indicate that females are more prone to develop Frailty Syndrome^(25,26)^, as well as finding that elderly women presented a higher occurrence of weight loss, fatigue, muscle weakness and low level of physical activity^(23)^. Additionally, females are 3 to 4.6 times more likely to develop frailty when compared to males^(27)^. The results presented here differed from a study with a sample group made up exclusively of elderly women, finding a higher prevalence in the group classified as frail paired with a significant increase in the vulnerable condition^(10)^, also highlighting that frailty compromises quality of life.

It was not possible to establish any association between age and EFE, which was in disagreement with other studies^(6,17,18)^ that reported a direct relationship between these variables.

Advances in frailty research attest to the influence of gender and age, as well as further conditions regarding the dynamics and living situation of elderly people. While phenotype mappings of the Brazilian elderly population has been the subject of some studies, a systematic review found this to still be a controversial area of investigation, due to the different lines of research and diverse protocols^(27)^. Regardless, there is a consensus that functional disability, risk of falls and negative self-perception of health were common elements in some studies.

As shown in Table 3, there was a significant association between these three tools. However, the relationship was weak and inverse between DGI-*Brazilian brief* and EFE, and moderate and positive between the latter and TUG, which was in line with other studies that presented similar findings^(4)^.

Overall, the highest levels of prevalence of falls were found in the frail elderly, which was already demonstrated in a previous study^(17)^.

Some studies, mainly those focused on systematic review^(21,22)^ proved the importance of TUG for identifying frail individuals within a community, but it was not able to differentiate frail from pre-frail patients, given that many false-positive results were reported, which could limit its applicability. This means that this method cannot be used in isolation as a testing tool to identify frailty. The combination of multiple protocols for any evaluation decreased the risk of false negative occurrence^(5)^. By contrast, a recent study^(27)^ showed that TUG was an effective tool in identifying frailty.

Studies^(25,26)^ demonstrate that elderly people categorized as non-frail presented a better performance in TUG than those classified as frail or pre-frail, which is consistent with the results analyzed here.

It increases the importance of the study on dynamic or functional balance and its relationship with frailty. The results of the meta-analysis^(28)^ emphasized that in the comparison of non-frail elderly people, those identified as frail showed the highest risk of falls, followed by pre-frail elderly. This study confirmed that frail elderly individuals were more likely to experience recurrent falls.

Convenience sampling can be considered a limitation of the present study. Furthermore, the predominance of females coupled with the low adherence of the male public may have contributed to the results obtained. No confounding factor analysis was performed for the dizziness complaint variable, since it was not the study object, and it is worth mentioning that the emergence of the Sars-Cov-2 pandemic hampered data collection.

It is clear that such findings, especially the increased risk of falls and the relevant occurrence of Frailty Syndrome in a socially active sample group, highlight the need to develop strategies for prevention and intervention for those 60 years of age and older, regardless of gender, longevity, and socioeconomic situation. There is unanimity among studies regarding the possible correlations between different tools and objectives, to better understand how one’s way of living can interfere with aging, favoring the onset of this syndrome and impacting the quality of life of the elderly^(29)^.

Understanding the various aspects that constitute balance and fragility can lead to an improvement in the functional difficulties that are important risk factors for falls in the elderly. These are issues that can be eliminated or resolved with educational and preventive action^(19)^, as long as elderly individuals are evaluated for possible geriatric syndromes such as frailty, which can be addressed to reduce the risk of bone fractures and death^(29)^. Therefore, early identification is crucial for the development of preventative strategies to slow the onset of further frailty stages^(30)^.

## CONCLUSION

Frailty and pre-frailty were identified in a significant portion of the volunteers, especially in the oldest elderly individuals. Functional and dynamic balance were moderately correlated with frailty, which demonstrated that Frailty Syndrome increases the risk of falls.
