# Supplementary material for: Frailty syndrome and risks for falling in the elderly community
Source: Codas. 2022 Aug 8;34(6):e20210025. doi: 10.1590/2317-1782/20212021025en (PMC9886293; doi:10.1590/2317-1782/20212021025en)
Supplement: Translation

# Translation: Síndrome da fragilidade e riscos para quedas em idosos da comunidade

**DOI:** 10.1590/2317-1782/20212021025pt

**Authors:** Carlos Kazuo Taguchi, Pedro de Lemos Menezes, Amanda Caroline Souza Melo, Leonardo Santos de Santana, Wesley Rayan Santos Conceição, Gabrielle Feitosa de Souza, Brenda Carla Lima Araújo, Allan Robert da Silva

**Affiliations:** 1 Universidade Federal de Sergipe – UFS - São Cristóvão (SE), Brasil.; 2 Universidade Estadual de Ciências da Saúde de Alagoas – UNCISAL - Maceió (AL), Brasil.

**Keywords:** Fragilidade, Idoso Fragilizado, Acidentes por Quedas, Envelhecimento Saudável, Idoso

## Abstract

**Objetivo:**

Identificar a prevalência da Síndrome da Fragilidade em idosos e suas relações com o risco para quedas.

**Método:**

Estudo clínico descritivo, transversal e analítico. Cento e um voluntários com mais de 60 anos, foram submetidos à avaliação audiológica, Dynamic Gait Index – Brazilian brief (DGI), Timed Up and Go(TUG) e Escala de Fragilidade de Edmonton (EFE) que determinaram, respectivamente, os limiares auditivos, síndrome da fragilidade, equilíbrio funcional e dinâmico e risco para quedas. Utilizou-se a distribuição percentual simples, o teste de Wilcoxon e de Correlação Bivariada com coeficiente de Pearson para a análise estatística. Foram adotados limites iguais inferiores a 1,0 e 5,0%.

**Resultados:**

A EFE identificou 22,8% dos voluntários como frágeis e 22,8% como vulneráveis. O DGI e o TUG classificaram 34,6 e 84,1% de riscos para quedas. Ocorreu correlação significativa entre a EFE e o DGI (p<0,01), a EFE e o TUG (p<0,01) e o DGI e TUG (p<0,01). O coeficiente de Pearson entre EFE e o DGI, entre o EFE e o TUG e DGI e TUG foram -0,26, -0,41 e 0,46 respectivamente. Ocorreu associação entre DGI e TUG e idade (p<0,01). Não houve correlação entre a EFE com sexo e idade.

**Conclusão:**

A fragilidade e pré-fragilidade foi identificada em uma parcela expressiva dos voluntários, sobretudo nos mais longevos. O equilíbrio funcional e o dinâmico se correlacionaram moderamente com fragilidade, o que demonstrou que a Síndrome da fragilidade aumenta o risco para quedas.

## INTRODUÇÃO

O progressivo crescimento da expectativa de vida da população é fato mundial, atual e irreversível. No Brasil o número de idosos passará de 19,2 milhões (9,2%) para 58,2 milhões (25,5%) em 2060, o que implicará no aumento da demanda por serviços de saúde voltada para esta população^(1)^. No envelhecimento ocorrem alterações fisiológicas, em maior ou menor intensidade, em todos os órgãos e sistemas^(2)^, o que acarreta na perda de autonomia e independência. Ainda, pode ocorrer a presença de maior susceptibilidade às doenças, declínio funcional e propensão para quedas. Este conjunto de sinais/sintomas é definido como a síndrome clínica de fragilidade^(3)^.

O mapeamento de risco para quedas pode ser realizado por diversos instrumentos de avaliação qualitativa como o *Dynamic Gait Index* (DGI) *Brazilian brief* ^(4)^, que avalia o equilíbrio dinâmico e a marcha em diferentes contextos, e o *Timed Up and Go* (TUG)^(5)^ que analisa o equilíbrio funcional. A fragilidade pode ser estudada por várias ferramentas, uma vez que não existe um padrão ouro, e que avaliam domínios e habilidades funcionais diversas e estabelecem nota de corte adaptado para as características de cada grupo populacional conforme demonstrado em estudo de revisão sistemática^(6)^.

Tanto os eventos de quedas, quanto a fragilidade do idoso são fenômenos passíveis de prevenção, de orientação e de encaminhamentos, o que torna o assunto prioritário quanto ao desenvolvimento de processos e serviços para os idosos^(7)^. Destaca-se que a Fonoaudiologia tem contribuído de maneira importante na produção do saber na sua inter-relação com a geriatria/gerontologia e, nesse sentido, enfatiza-se a necessidade do aumento de estudos sobre o envelhecimento para a definição de ações e campos de pesquisa na área^(8)^. Evidência recente aponta uma tendência à atuação na reabilitação e não somente nos aspectos de prevenção, e, ainda pontuou a área da Audiologia como a que menos contribuiu para esse conhecimento^(9)^.

Pesquisar a associação da fragilidade, marcha e equilíbrio dinâmico podem fornecer os critérios iniciais de ações com enfoque na atenção primária, na qual a Fonoaudiologia está inserida e com abrangência maior a ser ocupada na área da prevenção, educação e intervenção. Avanços científicos contribuem para o fortalecimento e consolidação desse saber, e, podem fortalecer as evidências de que a longevidade e a funcionalidade estão fortemente relacionadas com a instalação da fragilidade, bem como no seu agravo quando não são adotadas medidas de prevenção e intervenção^(10)^.

A partir da premissa que a fragilidade envolve a interação complexa de fatores biológicos, psicológicos e sociais ao longo da vida^(2)^, e que os efeitos desse processo podem se tornar irreversíveis, o objetivo deste estudo foi identificar a prevalência da Síndrome da Fragilidade em idosos e estabelecer suas relações com o risco para quedas.

## MÉTODO

Estudo clínico descritivo, transversal e analítico que foi desenvolvido no Laboratório de Audiologia e Equilíbrio (LAE), com voluntários selecionados nos ambulatórios de Otorrinolaringologia, Gastroenterologia e Cardiologia, do Hospital Universitário da instituição de origem e alunos da Universidade da Terceira Idade de instituição parceira. Projeto aprovado pelo Comitê de Ética e Pesquisa sob o protocolo 10266919.1.0000.

A composição da amostra, por conveniência, foi recrutada entre setembro de 2019 e março de 2020 na sala de espera dos consultórios das especialidades mencionadas, e em salas de aula da terceira idade, por meio de explanação e convite. Foram avaliados 101 voluntários, dos quais 80 do sexo feminino e 21 do masculino, todos com idade maior ou igual a 60 anos. Todos os participantes assinaram termo de consentimento livre e esclarecido.

Foram excluídos aqueles com perda auditiva neurossensorial de grau moderado a severo com comprometimento de compreensão da fala; presença de distúrbios neurológicos ou psiquiátricos que limitassem a execução das ordens dos instrumentos de avaliação; alteração estrutural de membros inferiores e que utilizam próteses ou órteses.

Como critério inclusivo, além da variável idade, foi realizada a avaliação audiológica básica com pesquisa do limiar tonal por via aérea e óssea, e logoaudiometria. Adotou-se os procedimentos e parâmetros consagrados na prática clínica^(11)^.

### Todos os participantes foram submetidos aos seguintes procedimentos:

1. Preenchimento de dados de identificação e sociodemográficos compostos por: idade; escolaridade; renda mensal; atividades de lazer; uso de medicamentos; queixa de tontura; quedas auto relatadas no último ano; tabagismo; etilismo; sintomatologia por outras comorbidades, e autoavaliação quanto ao grau de satisfação da sua saúde;
2. A Escala de Fragilidade de Edmonton (EFE) validado para o português do Brasil^(12)^ é composta por 11 itens em que o avaliador pode marcar três opções de diferentes respostas, em que a = zero (0), b = um (1) e c = dois (2) pontos. Foi obedecida esta sequência: 1) Avaliação da Cognição em que foi solicitado que o idoso desenhasse um relógio marcando 11h:10min; 2) Estado geral de saúde em que o idoso respondeu a pergunta nos últimos 12 meses, quantas vezes você foi internado (a)?; 3) Estado geral de saúde em que solicitou que o idoso descrevesse sua saúde de um modo geral; 4) Independência funcional em que o idoso respondeu em que atividades ele precisava de ajuda?; 5) Suporte social em que foi questionado quando precisa de ajuda, ou contar com a ajuda de alguém que aprove às suas necessidades?; 6) Uso de medicamentos em que foi questionado se normalmente ele usa cinco ou mais remédios diferentes e receitados (pelo médico); 7) Uso de medicamentos em que respondeu se esquece de tomar os seus remédios; 8) Nutrição em que foi perguntado se recentemente tem perdido peso, de forma que suas roupas estão mais folgadas; 9) Humor em que deve responder se sente triste ou deprimido(a) com frequência?; 10) Continência em que responde se tem problema de perder o controle da urina sem querer; 11) Desempenho funcional em que se realizou o teste *Timed Up and Go* – TUG^(5)^.

No TUG é avaliado o equilíbrio dinâmico no qual o tempo de realização de um trajeto delimitado foi cronometrado. Esse percurso consiste em sair da posição sentada, caminhar três metros, contornar um objeto estático, retornar e adotar a posição inicial. Quando o tempo dispendido for igual ou inferior a 10 segundos o voluntário é classificado com baixo risco, entre 11 e 20 segundos com médio risco e com tempo superior a 20 segundos com alto risco para quedas.

Foi adotado o teto máximo na EFE de 17 pontos, uma vez que os ítens de 6 a 10 computam até um e os demais até 2 pontos. Os resultados entre 0 e 4 pontos na EFE correspondeu à classificação não frágil; de 5 a 6 vulnerável ou pré-frágeis; de 7 a 8 fragilidade leve; de 9 a 10 moderada, e acima de 11 severa^(12)^.

1. Para avaliar o risco para quedas, os voluntários foram submetidos ao *Dynamic Gait Index*(DGI) – *Brazilian brief* ^(4)^, composto por cinco tarefas capazes de avaliar a marcha e o equilíbrio funcional. Conforme descrito no Quadro 1, neste teste é solicitado o deslocamento do voluntário em linha reta (cerca de três metros) em que se avalia a marcha em diferentes contextos sensoriais, como mudanças na velocidade, realização de movimentos horizontais e verticais da cabeça, giro sobre seu próprio eixo corporal e subir e descer escada. A variação da pontuação foi entre 0 e 3, em que 0 indica comprometimento grave, 1 comprometimento moderado, 2 comprometimento leve e 3 indica normalidade na execução da tarefa. A pontuação teto foi 15 pontos e, valor igual ou inferior a 11 pontos indicou alto risco para quedas.

**Quadro 1:** Descrição das cinco tarefas do Dynamic Gait Index – Brazilian brief (DGI)

Tarefa 1	*Marcha com movimentos horizontais (rotação) da cabeça*
Tarefa 2	*Marcha com movimentos verticais (rotação) da cabeça*
Tarefa 3	*Marcha e giro sobre o próprio eixo corporal (pivô)*
Tarefa 4	*Passar por cima do obstáculo*
Tarefa 5	*Subir e descer degraus*

No final da coleta, os dados foram analisados, e posteriormente, estabelecidas as possíveis correlações. Para a análise estatística foi considerada a variável dependente das respostas de cada usuário em cada uma das escalas. Foram adotadas as medidas descritivas com média, mediana e desvio padrão; e os testes de Wilcoxon e o de correlação bivariada com grau de relacionamento linear analisado por meio do coeficiente de Pearson com nível de significância em 0,01 e 0,05 destacados com * e ** respectivamente.

## RESULTADOS

Os resultados a seguir correspondem à análise dos dados de 101 idosos, com faixa etária entre 60 e 85 com média de 69,7 (±6,5) anos. Foram analisados os dados de 80 (79,2%) voluntários do sexo feminino e 21 (20,8%) do masculino (Tabela 1). Quarenta e seis idosos (45,5%) foram identificados como frágeis e vulneráveis, 38 (82,6%) pertenciam ao sexo feminino e 8 (17,4%) ao masculino. Dentre os que apresentaram fragilidade em algum grau, 12 (26,1%) apresentaram fragilidade leve; 7 (15,2%) moderada, e 4 (8,7%) com fragilidade severa.

**Tabela 1:** Distribuição da média, mediana e desvio padrão de idade dos 101 voluntários idosos avaliados segundo o sexo

Estatísticas	Masculino	Feminino	Geral
Amostra (n)	21	80	101
Média	67,6	69,0	69,7
Mediana	67	69	68
Desvio Padrão	6,0	6,6	6,5

Da aplicação do DGI, 34 (33,7%) dos voluntários apresentaram nota de corte dentro do risco de quedas. A variação obtida esteve entre 4 e 15 e média de 12,2 (±2,3) pontos conforme apresentada na Tabela 2. Quando considerado separadamente, o grupo com fragilidade, este percentual foi equivalente a 16 (36,9%) deles com riscos para quedas.

**Tabela 2:** Distribuição da média, mediana e desvio padrão dos resultados de 101 voluntários idosos segundo os testes *Dynamic Gait Index*-*Brazilian brief*, *Timed Up and Go* e Escala de Fragilidade de Edmonton

Estatísticas	DGI	TUG	EFE
Média	12,2	12,7	4,6
Mediana	13	12	4
Desvio Padrão	2,3	3,38	2,6

**Legenda:** DGI = *Dynamic Gait Index*-*Brazilian brief*; TUG = *Timed Up and Go*;

EFE = Escala de Fragilidade de Edmonton

Ainda na Tabela 2, observa-se que da aplicação do TUG, a variação foi de 7 a 26 com média de 12,7 (±2,6) segundos. Oitenta e cinco (84,1%) voluntários da amostra estiveram no risco de quedas, e a prevalência dentre os frágeis atingiu 100%.

Na EFE verificou-se resultados entre um 1 e 11 com média de 4,6 (±2,6) pontos, o que indicou uma variação de categorização de vulneráveis a frágeis severos. Vinte e três (22,8%) voluntários foram identificados como frágeis e o mesmo percentual com vulnerabilidade.

O teste de Wilcoxon indicou a ausência de correlação entre EFE e sexo (p=0,68), o que provou que esta variável separadamente não foi um preditor de fragilidade.

A incidência de quedas autorelatadas no último ano foi de 22,7% e naqueles com síndrome da fragilidade esse percentual atingiu 28,0%.

A Tabela 3 apresenta os resultados da aplicação do Teste de Correlação Bivariada com grau de relacionamento linear com coeficiente de Pearson. Ocorreu correlação significativa entre a escala de Edmonton e o DGI (p<0,01) e a escala de Edmonton e o TUG (p<0,01). Contudo, a correlação foi inversa e de fraca força, no primeiro caso (r=-0,26), e direta e de média força no segundo caso (0,47). O estudo estatístico revelou, ainda, correlação inversa e significativa entre o DGI e o TUG (p<0,01), com média força (r=-0,41).

**Tabela 3:** Resultados do teste de Correlação bivariada com coeficiente de Pearson segundo a Escala de Fragilidade de Edmonton, *Dynamic Gait Index*-*Brazilian brief* e *Timed Up and Go*

Correlação de Pearson	EFE	DGI	TUG
EFE	1	-0,26^*^	0,47*
DGI	-0,26*	1	-0,41*
TUG	0,47*	-0,41*	1

* correlação significativa no nível 0,01

**Legenda:** EFE = Escala de Fragilidade de Edmonton; DGI = *Dynamic Gait Index*-*Brazilian brief*;

TUG = *Timed Up and Go*

Na Tabela 4, verifica-se que, por meio da aplicação do Teste de Correlação Bivariada com grau de relacionamento linear com coeficiente de Pearson, foi observado que a idade correlacionou-se de maneira inversa, significativa e de média força com a DGI (p<0,01 e r=-0,33), e de maneira direta, significativa e de fraca força com a TUG (p=0,01 e r=0,18). Não foi encontrada associação de idade com a EFE. Foi observada a influência do TUG sobre os resultados que apontaram alteração na EFE com variados níveis de fragilidade. Houve associação significante (p<0,01) e correlação direta e de média força (r=0,47) entre estes dois instrumentos.

**Tabela 4:** Resultados do teste de Correlação bivariada com coeficiente de Pearson segundo a Idade, Escala de Fragilidade de Edmonton, Dynamic Gait Indes-*Brazilian brief* e *Timed Up and Go*

Correlação de Pearson	Idade	DGI	TUG	EFE
Idade	1	-0,33^*^	0,13^**^	-0,20
DGI	-0,33*	1	-0,41*	-0,26*
TUG	0,13**	-0,41*	1	-0,47*
EFE	0,20	-0,26*	-0,47*	1

* correlação significativa no nível 0,01

** correlação significativa no nível 0,05

**Legenda:** DGI = *Dynamic Gait Index*-*Brazilian brief;* TUG = *Timed Up and Go*; EFE = Escala de Fragilidade de Edmonton

## DISCUSSÃO

O DGI-*Brazilian brief* foi capaz de identificar que um terço da amostra pontuou abaixo da nota de corte, e que no TUG cerca de 20,0% da amostra apresentou baixo risco para quedas. Estes resultados estão em consonância com estudos recentes^(5,13)^ que provaram que o DGI identificou entre 30,0 e 40,0% dos idosos com alteração do equilíbrio funcional. Quanto ao desempenho no *Timed Up and Go*, os resultados obtidos estiveram de acordo com outros estudos^(13,14)^ que revelaram alteração entre 60,0% a 80,0% em idosos da comunidade.

Destaca-se que a utilização de dois instrumentos se mostrou positiva para estimar quedas futuras visto que a correlação moderada e negativa foi obtida conforme apresentado na Tabela 1, e ratificada em outro estudo^(4)^.

A destacada possibilidade de idosos mais longevos possuírem maior risco para quedas^(4,12)^ esteve em concordância com os achados aqui revelados em que a idade esteve moderada e fracamente correlacionada com o DGI-*Brazilian brief* e o TUG. O tempo menor dispendido no TUG sugeriu que idosos podem apresentar boa mobilidade funcional, entretanto, um número expressivo de indivíduos com valores mais elevados estiveram mais propenso às quedas, e aventou-se a possibilidade da existência concomitante de queixa de tontura e de desequilíbrios^(15)^, o que não foi analisado nesta pesquisa.

A prevalência da fragilidade obtida nesse estudo se mostrou similar ao de outros estudos que encontraram média de pontuação na EFE entre 25,9%^(16)^ e 30,9%^(17)^. Em contrapartida, a prevalência foi inferior a outro estudo que encontrou percentuais entre 40,5%^(18)^ e resultados mais divergentes foram obtidos com o mesmo instrumento, com percentuais entre 56,9%^(19)^ e 86%^(20)^.

Esta ampla variação de resultados foi reconhecida por estudo de revisão com metanálise^(6)^, que indicou uma variação de 6,7 a 44,0%, o que corroborou com os resultados apresentados. Outras pesquisas mostraram percentuais inferiores aos aqui demonstrado com variação entre 7%^(21)^ e 10,5%^(22)^, e que se justificaria pelas características de populações com melhores condições ambientais ou socioeconômicas, em que a longevidade esteve agregada à melhor qualidade de vida. A relação qualidade de vida está fortemente associada com fragilidade^.^ e pode ser impulsionada ou prejudicada conforme o meio socioeconômico ou situação social do idoso. Revela-se que há uma parcela importante de idosos que estão na situação de pré-fragilidade^(21,22)^ e se inclui na faixa populacional que deve ser identificada precocemente, visto que possuem risco aumentado para a fragilidade em torno de 13 a 31%^(11)^. Destaca-se que esta é uma situação preocupante pois indica algum processo em instalação, o que acarreta a necessidade de intervenção com o objetivo de impedir ou retardar a fragilização e a possível perda da capacidade funcional^(23)^.

A prevalência de auto relato de quedas pregressas neste estudo foi inferior a de outros estudos que indicaram percentuais mais elevados de até 51,7%^(24)^.

Apesar da ausência da existência da relação de quedas pregressas e a Síndrome da Fragilidade, pesquisas mostraram associação entre estas variáveis^(20,24)^ e, ratificada por resultados próximo ao obtido nesse estudo^(6)^.

A incidência de 28,% de risco para quedas em idosos e associação com a Síndrome da Fragilidade em 74,2% colaboraram para exemplificar a associação entre queda e fragilidade, como também a possibilidade da ocorrência de bidirecionalidade que é o fato da queda tornar o idoso frágil, do mesmo modo que a fragilidade promove eventos de queda^(18)^.

Como comentado, a Fonoaudiologia não vislumbrou a Síndrome da Fragilidade como potencial objeto de estudo, o que o torna-se urgente e iminente essa necessidade, uma vez que o envelhecimento populacional é fato irreversível e atual. Medidas nos três níveis de atenção à saúde do idoso devem ser desenvolvidas a partir de evidências científicas que revelam que a fragilidade é um preditor para risco de queda^(24)^.

A ausência de correlação entre EFE e sexo discordou de revisões que apontaram que o sexo feminino está mais propenso ao desenvolvimento da Síndrome da Fragilidade^(25,26)^ e foi demonstrado que idosas apresentaram maior frequência de perda de peso, fadiga, fraqueza muscular e baixo nível de atividade física^(23)^, ainda mais, de 3 a 4,6 vezes mais chances de desenvolver fragilidade quando comparado ao sexo masculino^(27)^. Os resultados aqui apresentados foram divergentes de estudo com uma amostra exclusivamente composta por idosas que apontou a prevalência maior na faixa classificada como frágil e um aumento importante na condição vulnerável^(10)^, e destacou que a fragilidade compromete a qualidade de vida.

Não foi possível estabelecer quaisquer associações entre idade e EFE, o que foi dissonante de estudos^(6,17,18)^ que certificaram a relação direta entre essas variáveis.

Apesar dos avanços, no estudo da fragilidade, atestarem a influência do sexo e idade, bem como as várias condições que envolvem a dinâmica e situação de vida dos idosos, admite-se que a intenção da constituição do fenótipo de idosos brasileiros foi objeto de alguns estudos, porém, em revisão sistemática foi comprovado que é escopo de difícil consenso em função das diferentes correntes de estudo e diversidade de protocolos^(27)^. Mas, por outro lado, houve a consonância que a incapacidade funcional, o risco de queda e a autopercepção de saúde negativa foram elementos visualizados em algumas investigações.

Conforme demonstrada na Tabela 3, ocorreu associação significante entre os três instrumentos. Porém, a relação foi fraca e inversa entre o DGI-*Brazilian brief* e EFE, e moderada e positiva entre esta última e o TUG, o que foi consonante com estudo que apresentou valores similares^(4)^.

De uma forma geral, os níveis mais elevados de prevalência de queda foram encontrados em idosos frágeis, o que fora demonstrado em estudo anterior^(17)^.

Algumas pesquisas, principalmente, aquelas voltadas para a revisão sistemática^(21,22)^ provaram a relevância do TUG para destacar os sujeitos frágeis dentro de uma comunidade, porém não foi capaz de diferenciar os indivíduos frágeis dos pré-frágeis, pois implicou em muitos resultados falso-positivos, o que restringiria sua aplicação. Isto significa que esse instrumento não pode ser usado ​​como teste único para identificar a fragilidade. A junção de mais protocolos para qualquer avaliação diminuiu o risco da ocorrência de falso negativo^(5)^. Em contrapartida, um estudo recente^(27)^ apontou que o TUG foi instrumento eficiente na identificação de fragilidade.

Estudos^(25,26)^ certificaram que idosos categorizados como não frágeis apresentaram tempo de execução no TUG menor que os de grupos frágeis ou pré-frágeis, e que foi consonante com os resultados aqui analisados.

Agrega-se à importância do estudo sobre equilíbrio dinâmico ou funcional e suas relações com a fragilidade. Os resultados da meta-análise^(28)^ sublinharam que na comparação de idosos não frágeis, aqueles identificados como frágeis demonstraram o maior risco de quedas, seguidos por idosos pré-frágeis. Este estudo confirmou que os idosos frágeis estiveram mais propensos a sofrer quedas recorrentes.

A amostra por conveniência, pode ser considerada uma limitação deste estudo. Além disso, o fato da predominância do sexo feminino e da pouca adesão do público masculino pode ter contribuído para os resultados encontrados. Não foi realizada a análise de fator de confusão para a variável queixa de tontura, uma vez que não foi objeto desse estudo, e cabe destacar o surgimento da pandemia pelo Sars-Cov-2 que dificultou a coleta de dados.

Ressalta-se que os achados encontrados, principalmente o risco aumentado para quedas e a importante ocorrência da Síndrome da Fragilidade numa amostra socialmente ativa evidencia a necessidade de empregar políticas para o planejamento de estratégias de prevenção e intervenção em todos aqueles que chegam aos 60 anos, independente de sexo, longevidade e situação socioeconômica. Concorda-se com o estudo que reforça as possíveis correlações entre diferentes instrumentos e objetivos para entender melhor como a forma de viver pode interferir na maneira de envelhecer, e favorecer a instalação dessa síndrome que pode impactar a qualidade de vida do idoso^(29)^.

Compreender as diversas faces que integram o equilíbrio e fragilidade podem desencadear um processo para melhorar as dificuldades de funcionalidade que são importantes fatores de risco para quedas em idosos. São questões que podem ser eliminadas ou dirimidas com ação educativa e preventiva^(19)^, e que, os idosos devem ser avaliados quanto à possibilidade de instalação de síndromes geriátricas, como a fragilidade, que podem ser abordadas para reduzir o risco de fraturas ósseas e morte^(29)^. Portanto, essa identificação precoce é crucial para elaboração de estratégias de intervenção antecipatória ao acometimento de outros níveis de fragilidade^(30)^.

## CONCLUSÃO

A fragilidade e pré-fragilidade foi identificada em uma parcela expressiva dos voluntários, sobretudo nos mais longevos. O equilíbrio funcional e o dinâmico se correlacionaram moderadamente com fragilidade, o que demonstrou que a Síndrome da fragilidade aumenta o risco para quedas.
